# Recurrent Neuroendocrine Tumor of the Cervix Presenting With Ectopic Cushing’s Syndrome

**DOI:** 10.7759/cureus.31541

**Published:** 2022-11-15

**Authors:** Jeffrey G Rico, Nadim Bou Zgheib, Sheena Pramrod, Omolola Olajide

**Affiliations:** 1 Department of Medicine, West Virginia University School of Medicine, Morgantown, USA; 2 Department of Obstetrics and Gynecology, Marshall University Joan C. Edwards School of Medicine, Huntington, USA; 3 Nephrology, University of Florida College of Medicine, Jacksonville, USA; 4 Department of Endocrinology, Marshall University Joan C. Edwards School of Medicine, Huntington, USA

**Keywords:** ectopic, cervix, carcinoma, neuroendocrine tumor, cushing’s syndrome

## Abstract

Neuroendocrine carcinomas (NEC) of the cervix are a rare disease entity and account for only 1-2% of cervical carcinomas. The small-cell variant is the most common, with a worse prognosis and a higher rate of lymphatic and hematogenous metastases when compared with other subtypes of NEC. The diagnosis is usually made when the extra-pelvic disease is already apparent. Cushing’s syndrome due to adrenocorticotropic hormone (ACTH)-secreting tumors of the cervix is exceedingly rare. To date, there have been no reported cases in the literature of Cushing’s syndrome induced by the recurrence of metastases years after the initial diagnosis. This is a case of recurrent small-cell neuroendocrine carcinoma of the cervix presenting with Cushing’s syndrome five years after her original diagnosis. We present here the workup, management, and follow-up of this patient, including multisystemic, coordinated medical care.

## Introduction

Neuroendocrine carcinomas (NECs) are heterogenous groups of tumors derived from neuroendocrine cells. NECs of the cervix are rare and account for 1-2% of all cervical carcinomas, with the small-cell variant being the most common [1,2]. Small-cell NECs have a high rate of lymphatic and hematogenous metastasis even when the carcinoma is limited to the cervix. Patients usually present at a late stage, with the extra-pelvic disease being apparent at the time of diagnosis [2]. Among the different histologic variants of NEC of the cervix, the small-cell variant has the highest rate of recurrence [3]. Adrenocorticotropic hormone (ACTH)-secreting tumors of the cervix are rare [4]. We present a case of recurrent metastatic NEC of the cervix five years after the original diagnosis of NEC of the cervix, now presenting with Cushing’s syndrome [1,2].

## Case presentation

A 39-year-old female with a history of recurrent small-cell cervical cancer presented to the emergency department (ED) of our hospital with complaints of weight gain, generalized facial edema, lightheadedness, tingling sensation of her entire face, bilateral leg edema, and abdominal distention.

**Figure 1:**
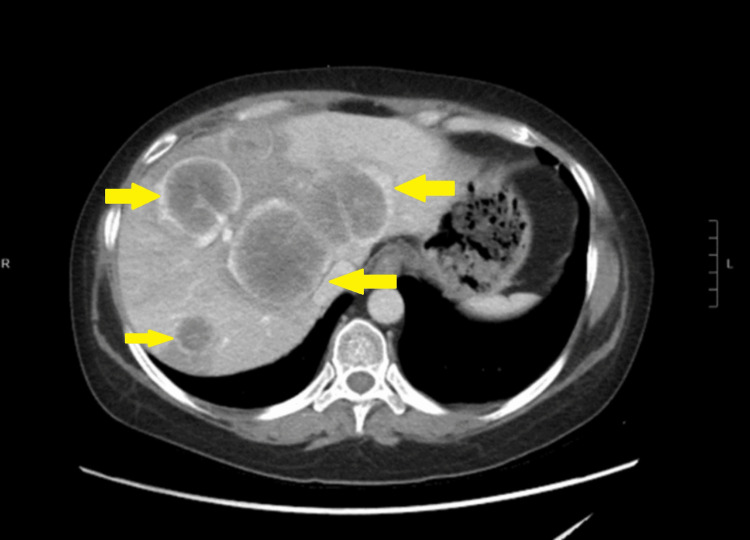
Abdomial CT with contrast done one month prior showed evidence of metastatic disease including multiple large liver lesions.

**Figure 2:**
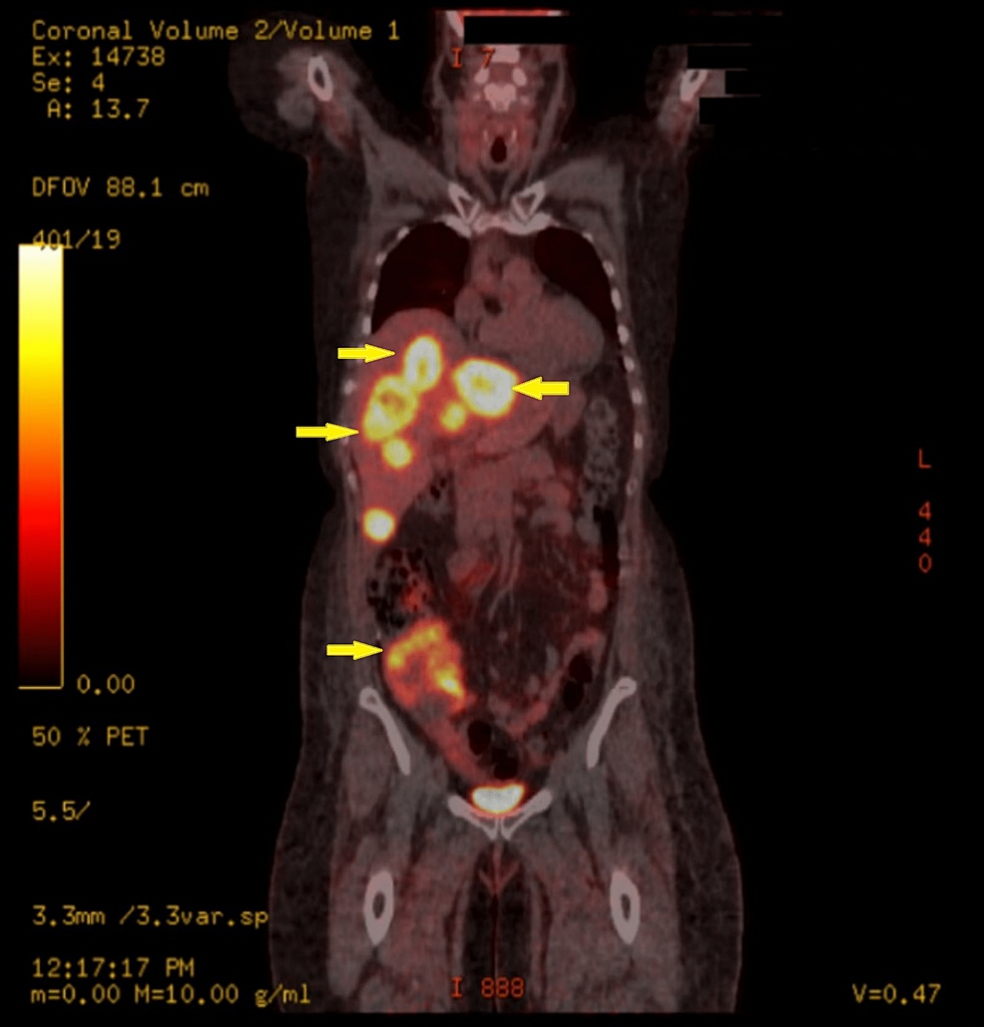
PET/CT demonstrated the presence of hypermetabolic lesions in the liver and right lower quadrant of the abdomen.

Her problems started a month prior to her ED visit, when she started to complain of abdominal distention. She had a computed tomography (CT) abdomen with contrast, which revealed evidence of metastatic disease, including multiple large liver lesions (Figure 1). Subsequently, she had a positron emission tomography (PET) scan, which confirmed the presence of hypermetabolic lesions in the right peritonsillar tissue, liver, right lower quadrant of the abdomen, and bilateral pulmonary nodules with lymphadenopathy in the left hilum (Figure 2). A liver biopsy was done, with the final pathology consistent with recurrent NEC of the cervix. She was started on cisplatin, etoposide, and atezolizumab by gynecologic oncology but started to develop facial swelling and progressive abdominal distention, prompting this ED consult and subsequent admission.

She had a significant medical history of being diagnosed with cervical cancer (FIGO stage 1B2 NEC) five years prior by gynecologic oncology, at which time she underwent concurrent chemo-radiation followed by surgical assessment of her pelvic lymph nodes with robotic pelvic lymph node dissection and bilateral ovarian transposition to avoid premature menopause. She was subsequently treated with cisplatin and pelvic radiation. She had a follow-up cervical biopsy several months after chemotherapy, which showed persistent NEC, but her PET scan showed no evidence of metastatic disease. After undergoing a robotic total laparoscopic hysterectomy, the final pathology showed a persistent microscopic focus of NEC of the cervix with negative margins. She received adjuvant chemotherapy with cisplatin and etoposide for six cycles with regular follow-up pap smears and annual PET scans, with no evidence of recurrence for five years.

On admission, her vital signs were: blood pressure = 129/79 mm Hg, pulse rate = 85/min, respiratory rate = 18/min, and temperature = 98.5 °F (36.9 °C). Her physical examination was notable for moon facies (a noticeable change from her pictures as recent as two months prior), supraclavicular and dorsocervical fat pads, multiple bruises on her arms, edema of her face and legs, acne of her face and neck, and hair growth of her chin area. No purple striae were seen on the abdomen.

Laboratory tests revealed leukopenia and thrombocytopenia (which were attributed to her chemotherapy), recently diagnosed diabetes (occasional hyperglycemia and HbA1c 7.7%), and electrolyte imbalances (hypokalemia and hypophosphatemia) (Table 1).

**Table 1:** Initial laboratory work showed leukopenia, thrombocytopenia, hyperglycemia, hypokalemia, and hypophosphatemia. AST: aspartate aminotransferase, CO_2_: carbon dioxide, BUN: blood urea nitrogen, ALT: alanine aminotransferase.

Sodium	142 mEq/L (135–145 mEq/L)
Potassium	2.0 mEq/L (3.5–5.0 mEq/L)
Chloride	98 mEq/L (98–108 mEq/L)
CO_2_	35 mEq/L (21–32 mEq/L)
Anion gap	9 mEq/L (8–16 mEq/L)
BUN	14 mg/dL (7–13 mEq/L)
Creatinine	1.13 mg/dL (0.6–1.1 mg/dL)
Glucose	460 mg/dL (74–100 mg/dL)
Calcium	7.8 mg/dL (8.5–10.1 mg/dL)
Phosphorous	1.0 mg/dL (2.5–4.5 mg/dL)
Albumin	2.5 mg/dL (3.1–4.5 mg/dL)
AST	43 U/L (15–27 U/L)
ALT	76 U/L (12–78 U/L)
White blood cell count	0.6 k/cmm (4.5–10.0 k/cmm)
Red blood cell count	3.55 million cells/μL (3.7–5 × 2)
Hemoglobin	11.9 g/dL (12.0–16.0)
Hematocrit	34.3% (35.0–47.0)
Platelet	45 k/cmm (150–440 k/cmm)

**Figure 3:**
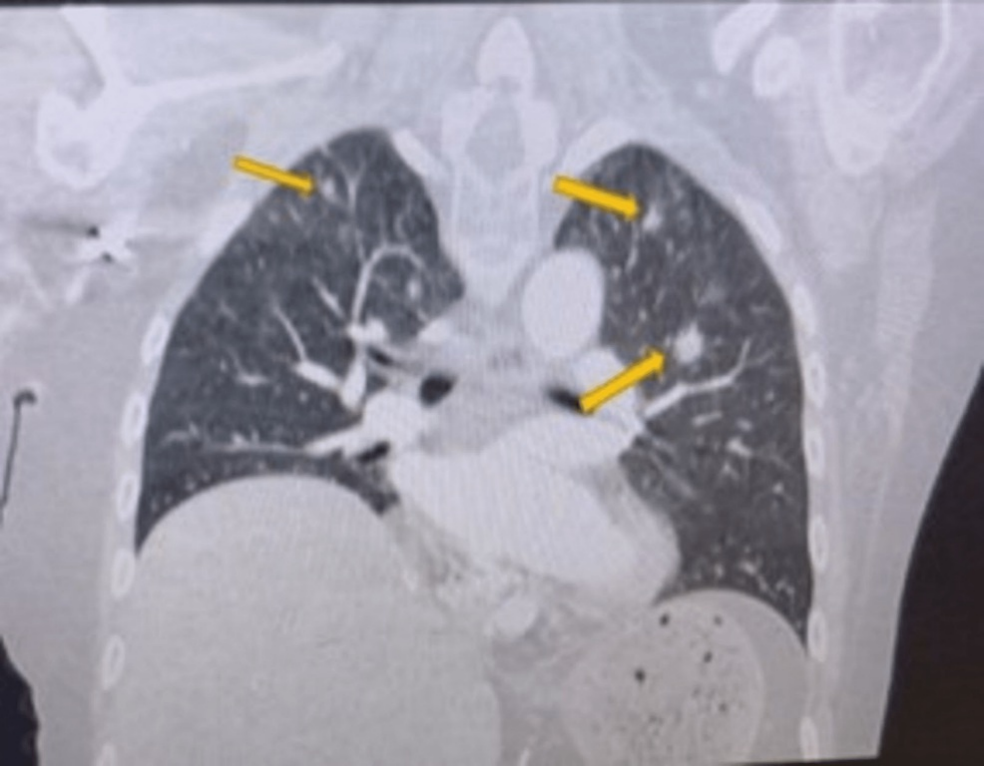
Contrast-enhanced chest CT showing bilateral noncalcified lung nodules.

Her chest X-ray showed bilateral pleural effusions. Magnetic resonance imaging (MRI) of the brain showed no evidence of pituitary masses, abnormalities, or metastatic disease in the brain. A CT of the chest showed new bilateral non-calcified lung nodules when compared to the previous PET scan, pathologic-sized left hilar adenopathy, and multiple peripherally enhancing hepatic nodules and masses (Figure 3). The adrenal glands were unremarkable. Workup for facial swelling and bilateral leg edema showed no evidence of superior vena cava (SVC) syndrome on both her chest CT and transthoracic echocardiogram.

She was admitted to the intensive care unit (ICU) and started on empiric antibiotics and filgrastim for neutropenia. Replacement therapy for both hypokalemia and hypophosphatemia was given. After both electrolytes were normalized, the patient was started on basal-bolus insulin therapy.

Based on her clinic presentation of excessive weight gain, new-onset hyperglycemia, hypertension with hypokalemia, and a history of NEC, suspicion of Cushing’s syndrome was high. Further workup showed elevated serum cortisol after 1 mg overnight dexamethasone suppression, elevated 24-hour urine cortisol, and elevated midnight salivary cortisol, which confirmed Cushing’s syndrome (Table 2). ACTH was also elevated, but dehydroepiandrosterone sulfate (DHEAS) was normal. Thyroid function tests showed a slightly low free thyroxine, but this was attributed to an acute illness.

**Table 2:** Work up showed elevated ACTH, elevated 24-hour urine cortisol, elevated salivary cortisol, and elevated serum cortisol after 1 mg overnight dexamethasone suppression test. HgbA1C: hemoglobin A1C; ACTH: adrenocorticotropic hormone; DHEAS: dehydroepiandrosterone sulfate; TSH: thyroid stimulating hormone; free T4: free thyroxine.

HgbA1C	7.7%	(4.0-6.0%)
ACTH	1207 pg/mL	(7.2–63.3 pg/mL)
24-hour urine cortisol	7070 μg/24 hr	(6–42 μg/24 hr)
Salivary cortisol	>1.000 μg /dL	(0.025–0.600 μg/dL)
Serum cortisol after 1 mg overnight dexamethasone suppression	143.0 μg/dL	(3.1–16.7 μg/dL)
Total testosterone	77 ng/dL	(14–76 ng/dL)
DHEAS	250.0 μg/dL	(57.3–279.2 μg/dL)
Chromogranin A	970.9 ng/mL	(0.0–101.8 ng/mL)
TSH	0.572 mIU/L	(0.358–3.74mIU/L)
Free T4	0.70 ng/dl	(0.76–1.46) ng/dl

A diagnosis of Cushing's syndrome due to metastatic small-cell neuroendocrine carcinoma of the cervix was assumed. A bilateral adrenalectomy, which is the definitive treatment of hypercortisolism when surgical removal of the source of excess ACTH is done, was not done because gynecologic oncology wanted to treat her with chemotherapy urgently due to her metastases and the nature of the disease and felt that surgery and recovery would delay the start of chemotherapy. Ketoconazole was felt to be a poor choice in the setting of liver metastases with worsening liver function tests. The patient was thus started on mifepristone 300 mg daily, as it is indicated for hypercortisolism secondary to endogenous Cushing’s syndrome with diabetes. Nephrology was consulted, and potassium supplementation was transitioned to oral potassium chloride 40 meq tablets four times a day; spironolactone 50 mg twice daily was added for the hypokalemia and hypertension, which occurred after the patient started bevacizumab. Hypokalemia is a common side effect of mifepristone therapy due to the glucocorticoid receptor blockade, which leads to cortisol's spillover effect on unopposed mineralocorticoid receptors. She was discharged home with a basal-bolus insulin regimen.

Her posthospitalization course was complicated by compression fractures of her lumbar spine one week after discharge with no history of falls. An MRI of the spine showed chronic compression fractures of the T11-L3 vertebral bodies with no evidence of osseous metastatic disease. Dual-energy X-ray absorptiometry (DXA) scan interpretation demonstrated osteoporosis. Vertebral fracture assessment showed morphometric fractures in the lower thoracic and upper lumbar vertebrae. She was subsequently treated with IV administration of 5 mg of zoledronic acid. She was also readmitted multiple times after her initial admission due to the patient's developing neutropenic fever, which was treated with filgrastim and antibiotics.

After starting mifepristone, her glycemic control improved to the point that insulin therapy could be subsequently discontinued. Her liver enzymes normalized, and ketoconazole was subsequently added for adjunct therapy to treat hypercortisolism, but the dose could not be optimized due to persistently elevated liver function tests. Hypokalemia management and resistant hypertension were additional challenges encountered by this patient.

At her follow-up visits, she had notably lost weight with the improvement of her leg edema. She continued to follow up with a nephrologist on an outpatient basis, and her normal potassium levels were normal on 40 meq of oral potassium chloride tablets four times a day and spironolactone 150 mg twice a day. She was followed up closely by her gynecologic oncologist and was on bevacizumab, topotecan, and paclitaxel before her unfortunate demise a few months later.

## Discussion

Cushing’s syndrome due to ectopic ACTH secretion only represents 9-18% of cases. Most primary endocrine tumors responsible for ectopic ACTH secretion are located in the chest [5]. Abdominal and retroperitoneal neuroendocrine tumors are the second- and third-most reported sites [5]. Neuroendocrine tumors of the cervix are incredibly rare [6-9].

A unique feature of this case is that the patient presented with Cushing’s syndrome due to neuroendocrine tumor metastases found five years after the primary site of the tumor was resected. For this patient, a biopsy of the liver confirmed a metastatic neuroendocrine tumor, but it is unknown if the other sites of metastases are implicated in the production of excess ACTH.

The management of this disease focuses on controlling hypercortisolism, consequent hyperglycemia, and hypokalemia. Surgical excision of ACTH-secreting neuroendocrine tumors is the most effective, but in cases where that is not possible, bilateral adrenalectomy and medical treatment are the next best treatments for this disease entity [10]. For this patient, bilateral adrenalectomy was not done as gynecologic oncology wanted to treat her with chemotherapy urgently due to the metastases and nature of the disease and felt that surgery and recovery would delay the start of chemotherapy.

We provided medical management for the patient’s hypercortisolism. Pharmacological therapy for hypercortisolism can be categorized into immediate-acting steroidogenesis inhibitors (metyrapone, ketoconazole, and etomidate), slow-acting cortisol-lowering drugs (mitotane), and glucocorticoid receptor antagonists (mifepristone) [5]. We initially chose mifepristone because it is indicated in patients with type 2 diabetes mellitus and could be given safely despite the patient’s worsening liver function levels [11].

As demonstrated, the management of recurrent hypokalemia proved challenging in this patient. The phenomenon is well known to be induced by ectopic ACTH. Several mechanisms contribute to this. Activation of renal tubular type 1 (mineralocorticoid) receptors by cortisol is thought to be the mechanism that applies mainly to patients with severe hypercortisolism due to ectopic ACTH secretion. Additionally, there may also be an increase in the production of renin substrate from the liver. The high serum cortisol concentrations may not be completely inactivated by 11β-hydroxysteroid dehydrogenase type 2 in the kidney and overwhelm its ability to convert cortisol to cortisone, resulting in activation of mineralocorticoid receptors resulting in potassium loss in the distal tubules [12]. Hypokalemia may also result from adrenal hypersecretion of mineralocorticoids, such as deoxycorticosterone and corticosterone. This can also be amplified by mifepristone, as it is a glucocorticoid receptor antagonist that increases circulating cortisol levels [12].

Complications such as hypokalemia, hyperglycemia, acute respiratory distress syndrome, infections, muscle wasting, hypertension, and bone fractures can occur and can arise at any time throughout the course of the disease when urine-free cortisol is fivefold or more above the upper limit of normal [5]. Ketoconazole was initially considered for medical treatment, but due to mildly elevated liver enzymes during the initial presentation, we decided to use mifepristone instead. A small cohort study showed that severe hypercortisolism and increased baseline transaminase levels could be due to cortisol-induced hepatic steatosis [13]. Later in her course, ketoconazole was added to her mifepristone therapy to decrease adrenal cortisol production. Unfortunately, her dose could not be increased due to the patient's persistently elevated liver enzymes.

Recurrent pancytopenia due to chemotherapy contributed to the protracted nature of this patient’s clinical course. Due to cortisol's immunosuppressive and anti-inflammatory effects, opportunistic infections can arise [14]. Since her initial hospitalization, she has been readmitted several times due to neutropenic fever, which was treated with filgrastim and antibiotics.

## Conclusions

Ectopic Cushing’s syndrome due to metastatic neuroendocrine small-cell carcinoma is a rare condition with a poor prognosis. The options for treatment are few and not necessarily curative. There needs to be increased awareness of this serious and rare complication. Managing the condition can be a challenge and requires a multidisciplinary team approach to improve outcomes.
